# Sustained improvement in work outcomes in employed patients with rheumatoid arthritis during 2 years of adalimumab therapy: an observational cohort study

**DOI:** 10.1007/s10067-020-05038-y

**Published:** 2020-03-23

**Authors:** Frank Behrens, Hans-Peter Tony, Michaela Koehm, Eva C. Schwaneck, Holger Gnann, Gerd Greger, Harald Burkhardt, Marc Schmalzing

**Affiliations:** 1 Division of Rheumatology, University Hospital Frankfurt, Goethe University, Frankfurt am Main, Germany; 2 Project Group Translational Medicine & Pharmacology TMP, Fraunhofer Institute for Molecular Biology and Applied Ecology IME, Frankfurt am Main, Germany; 3 Schwerpunkt Rheumatologie/Klinische Immunologie Medizinische Klinik und Poliklinik II, Universität Würzburg, Oberdürrbacher Strasse 6, Würzburg, Germany; 4 Abteilung Biostatistik, GKM Gesellschaft für Therapieforschung mbH, Munich, Germany; 5 AbbVie Deutschland GmbH & Co. KG, Wiesbaden, Germany

**Keywords:** Absenteeism, Adalimumab, Presenteeism, Rheumatoid arthritis, Therapeutic effects, Work performance

## Abstract

**Objective:**

The goal of this study was to evaluate the long-term impact of adalimumab therapy on work-related outcomes in employed patients with rheumatoid arthritis (RA).

**Method:**

We utilized data from an observational cohort of German patients who initiated adalimumab treatment during routine clinical care. Analyses were based on employed patients (part-time or full-time) who continued adalimumab treatment for 24 months. Major outcomes were self-reported sick leave days in the previous 6 months, absenteeism, presenteeism, and total work productivity impairment as assessed by the Work Productivity and Activity Impairment (WPAI) questionnaire and disease activity assessments. The normal number of sick leave days was based on data from the German Federal Statistical Office.

**Results:**

Of 783 patients, 72.3% were women, mean age was 47.9 years, and mean disease duration was 7.8 years. At baseline (before adalimumab initiation), 42.9% of patients had higher than normal sick leave days (> 5) in the previous 6 months. During 24 months of adalimumab treatment, 61% of patients with higher than normal sick leave days at baseline returned to normal sick leave values (≤ 5 days/6 months). Overall, mean sick leave days/6 months decreased from 14.8 days at baseline to 7.4 days at month 24. Improvements were observed in WPAI assessments and disease activity measures, although presenteeism levels remained high (32.2% at month 24).

**Conclusions:**

Adalimumab treatment was associated with strong and sustained improvements in work-related outcomes in employed patients who continued on adalimumab for 24 months. Presenteeism appears to be the work outcome most resistant to improvement during RA treatment.

**Trial registration:**

NCT01076205

**Table:** 

**Key Points** *• Long-term adalimumab therapy was associated with sustained improvements in work outcomes in patients with rheumatoid arthritis.* *• Despite improvements in sick leave days and work absenteeism, presenteeism (impairment while at work) remained relatively high.*

## Introduction

Rheumatoid arthritis (RA) is characterized by joint destruction and reductions in function and patient well-being [1], which can result in the reduced ability to engage in productive work [2–4]. Although loss of working time due to sick leave and disability is a key component of the effects of RA on employment, equally important is reduced productivity during days at work [5]. The impact of RA on working time and productivity is associated with large societal costs [6–8].

Effective RA therapy has been shown to improve work-related outcomes, including sick leave absences and productivity [5]. The magnitude of the benefit depends on various factors, including patient baseline characteristics, such as functional ability and disease duration, and country of residence [9, 10]. Recent studies on the effect of therapeutic intervention on work productivity have examined work-related outcomes over a period of 6 to 12 months [11–15] or have focused on patients with specific characteristics, such as early RA [13, 14, 16]. There is thus a need to further characterize the effects of longer-term treatment, particularly in patients with extended disease durations. We used data from a large observational study to explore the impact of 24 months of adalimumab therapy on sick leave days and work productivity in employed patients with RA during routine clinical care in Germany.

## Materials and methods

### Study design

This study utilized data from German patients with RA enrolled in a multicenter observational trial who received adalimumab therapy at the decision of the clinician (Clinicaltrials.gov trial registration NCT01076205). This report is based on interim data for the first 24 months of therapy. Patients included in these analyses were treated between September 15, 2009, and June 29, 2017.

Adult patients (≥ 18 years of age) were required to have a diagnosis of active RA, a clinical indication for treatment with a tumor necrosis factor inhibitor, and no contraindications. To be included in the analyses reported here, patients were required to have active disease (Disease Activity Score-28 joints [DAS28] ≥ 3.2), be employed full-time (≥ 35 h/week) or part-time at baseline (before treatment initiation) and throughout the 24-month period, and have adequate data for analyses performed here, including DAS28 at baseline and data on missed work days due to illness (sick leave days) at baseline and month 24. Patients who discontinued treatment, were lost to follow-up before month 24, or were not employed throughout the study were not included in these analyses. We excluded patients who left employment during the 24 months of treatment, including those who retired or received disability pensions, to allow a consistent patient cohort in which we could conduct an in-depth analysis of long-term outcomes in employed patients. All patients were informed of the objectives of the observational study and gave written consent for their voluntary participation in the study and the anonymous use of personal data in statistical analyses. Ethics approval was obtained from the Ethics Commission of the Medical Department of Goethe University, Frankfurt am Main, Germany (number 122/09).

During the first 24 months of treatment, visits were scheduled at months 0, 3, 6, 12, and 24. Patients were not asked about missed sick leave at the month 3 visit; thus, data for this visit are not included here.

### Outcome measures

Data for this study were collected on a Case Report Form completed by the clinician; patient-reported outcomes were based on patient responses. The primary outcome measure was sick leave days at baseline and during 24 months of adalimumab therapy. In Germany, a certificate issued by a doctor is required if more than 3 consecutive days are missed due to illness; shorter absences generally do not require documentation. For visits conducted at months 0, 6, and 12, employed patients were asked (English translation): “In the last 6 months, have you received a sick leave certificate from a doctor?” If the answer was “yes,” patients reported the total number of days covered by the sick leave certificate(s). At month 24, patients were asked to list the number of doctor-certified sick leave days over the past 12 months (since their last visit); this value was divided by 2 to be comparable with previous reporting periods. Work productivity was assessed using the Work Productivity and Activity Impairment (WPAI): General Health questionnaire, a validated tool that assesses work time missed due to illness (absenteeism [% of missed work hours due to health problems in the past 7 days]), impairment at work (presenteeism, assessed on a visual analog scale ranging from 0% [no impairment] to 100% [complete impairment] in the past 7 days), and total work productivity impairment (an aggregate measure of both absenteeism and presenteeism calculated as absenteeism rate + [(1 − absenteeism rate) × presenteeism rate]) [17, 18].

Disease activity was assessed using DAS28 [19] and function was assessed using the Health Assessment Questionnaire-Disability Index (HAQ-DI) [20]; for both measures, higher scores indicate greater impairment. At each visit, patients provided self-assessments of pain, fatigue, and global health in the past 7 days on an 11-point categorical scale ranging from 0 (best) to 10 (worst).

### Data analysis

All available data were analyzed; data were not imputed. Initial analyses revealed that sick leave days were asymmetrically distributed (positively skewed) due to the large number of patients with no sick leave days at baseline. Because asymmetrical distribution can compromise interpretation of mean values, we conducted subgroup analyses by baseline sick leave days based on the mean number of sick leave days in the overall German population during the years this study was conducted (2010–2015), referred to as “normal” sick leave. According to the German Federal Statistical Office, the annual sick leave days in Germany during these years ranged from 9.3 to 10.00, with a mean of 9.55 per year, or 4.775 per 6 month period [21]. We rounded this number to 5 to create 2 subgroups: 0 to 5 days/6-month period (representing a normal number of sick leave days in the overall population) and > 5 sick leave days/6-month period (representing an increased number of sick leave days compared with the overall population).

Differences in baseline characteristics between the subgroups with ≤ 5 and > 5 missed working days at baseline were assessed using the *t* test; *p* < 0.05 was considered statistically significant. Stepwise and backward logistic regression analyses were used to identify baseline characteristics that predicted sick leave days at baseline and change in sick leave days from baseline to month 6. Thirty-six baseline characteristics, including demographic and disease characteristics, comorbidities, concomitant and prior therapy, and educational level, were included in regression models.

## Results

### Baseline characteristics

Because the goal of this study was to examine the impact of long-term adalimumab therapy on sick leave days and work productivity, the study cohort was limited to patients who were employed full-time or part-time at both baseline and month 24; older patients who had retired from the work force and those who were too disabled to work were not included in these analyses. Accordingly, baseline characteristics were specific to employed patients initiating biologic therapy.

The study cohort (*N* = 783) had baseline characteristics consistent with moderate to severe RA (Table 1). Mean disease duration was approximately 8 years and mean DAS28 was 4.9. At baseline, 70% of patients reported full-time employment and 30% were employed part-time. Part-time employees were almost exclusively women (227/235; 96.6%); only 7 of 217 men (3.2%) were employed part-time. A total of 336 patients (42.9%) reported a higher than normal number of sick leave days in the previous 6 months (> 5 days), while the remainder (447 patients; 57.1%) reported a normal number of sick leave days in the previous 6 months (0–5 days). Patients with > 5 days of sick leave in the previous 6 months had more severe disease than those with ≤ 5 days, as indicated by significant between-group differences in DAS28 and the patient-reported outcomes of HAQ-DI, pain, fatigue, and global health (all *p* < 0.01).

**Table 1:** Baseline characteristics

Characteristic	Sick leave days in the past 6 months		All patients (*N* = 783)
	0–5 (*n* = 447)	> 5 (*n* = 336)	
Female (%)	74.9	68.8	72.3
Age (year)	47.8 (9.5)	48.1 (8.5)	47.9 (9.1)
Body mass index (kg/m^2^)	26.2 (5.0)	26.7 (5.5)	26.4 (5.2)
Disease duration (year)	7.9 (6.8)	7.5 (7.1)	7.8 (7.0)
Prior biologic therapy (%)	18.1	17.3	17.8
Previous joint surgery (%)	11.7	17.9	14.4
Concomitant MTX treatment (%)	58.2	62.8	60.2
DAS28	4.8 (1.0)	5.1 (1.1)	4.9 (1.1)
Tender joint count	7.0 (5.4)	8.9 (6.2)	7.8 (5.8)
Swollen joint count	5.1 (4.5)	6.0 (5.2)	5.5 (4.9)
Pain^a^	5.2 (2.3)	6.0 (2.2)	5.6 (2.3)
Fatigue^a^	5.2 (2.7)	6.1 (2.4)	5.6 (2.7)
Patient global health^a^	5.5 (2.2)	6.0 (2.3)	5.7 (2.2)
Morning stiffness duration (min)	53.6 (66.7)	71.0 (74.3)	61.0 (70.5)
HAQ-DI	0.84 (0.60)	1.16 (0.63)	0.98 (0.63)
Employment (%)			
Full-time (≥ 35 h/week)	67.1	73.8	70.0
Part-time	32.9	26.2	30.0
Sick leave days in past 6 months	0.5 (1.4)	33.7 (37.8)	14.8 (29.7)
WPAI absenteeism in past 7 days (% of hours)	8.1 (21.6)	34.1 (42.3)	19.2 (34.6)
WPAI presenteeism in past 7 days (%)	45.2 (24.2)	56.9 (25.3)	49.9 (25.3)
WPAI total work productivity impairment in past 7 days (%)	47.5 (25.4)	66.0 (25.9)	54.8 (27.1)

Data are reported as mean (standard deviation) unless otherwise indicated

*DAS28* Disease Activity Score-28 joints, *HAQ-DI* Health Assessment Questionnaire-Disability Index, *MTX* methotrexate, *WPAI* Work Productivity and Activity Impairment

^a^Assessed on an 11-point categorical scale from 0 (best) to 10 (worst)

Due to the study design, which required patients to have data on sick leave days at both baseline and month 24, patients who discontinued the study were not included. Information on discontinuation in the cohort of patients employed at baseline is presented in Online Resource 1.

Sickness absences based on recall over the previous 6 months were supported by the WPAI absenteeism assessment, which is based on the patient’s recollection of missed work hours due to illness in the past 7 days. The subgroup with > 5 sick leave days in the previous 6 months had a substantially higher rate of absenteeism than the subgroup with ≤ 5 days of sick leave (34.1% versus 8.1% of hours). Presenteeism (percentage impairment while at work) was also higher in the subgroup with > 5 compared with ≤ 5 days of sick leave at baseline (56.9% versus 45.2%).

### Change in work productivity during adalimumab therapy

In the full patient cohort, the mean (median) number of sick leave days in the past 6 months decreased during adalimumab treatment from 14.8 (2.0) at baseline to 7.4 (0.0) at month 24 (Table 2). The data were positively skewed toward lower values due to the large number of patients with no sick leave days at baseline (381/782; 48.7%). Because of the asymmetric data distribution, our primary analysis was based on the percentages of patients with sick leave days within or above the normal range rather than on mean values, which can be misleading with skewed data (see “Data analysis”). The proportion of patients with > 5 days of sick leave in the prior 6 months decreased from 42.9% (336/783) at baseline to 27.6% (216/783) at month 24.

**Table 2:** Change in work productivity during adalimumab therapy in employed patients

Outcome	Patient population	*n*^a^	Month			
			0	6	12	24
Sick leave days in past 6 months	All patients	783	14.8 (29.7)	8.8 (25.3)	10.0 (28.8)	7.4 (19.0)
	0–5 sick leave days at baseline	447	0.5 (1.4)	3.1 (9.7)	4.7 (17.7)	4.6 (13.6)
	> 5 sick leave days at baseline	336	33.7 (37.8)	16.5 (35.8)	17.5 (38.1)	11.1 (23.8)
WPAI absenteeism in past 7 days, % of hours	All patients	580	19.2 (34.6)	9.6 (25.7)	7.8 (23.3)	8.4 (23.1)
	0–5 sick leave days at baseline	332	8.1 (21.6)	5.4 (18.7)	5.6 (20.4)	6.1 (19.4)
	> 5 sick leave days at baseline	248	34.1 (42.3)	15.1 (32.0)	11.0 (26.6)	11.3 (26.8)
WPAI presenteeism in past 7 days, %	All patients	694	49.9 (25.3)	32.7 (22.6)	31.2 (22.5)	32.2 (23.0)
	0–5 sick leave days at baseline	416	45.2 (24.2)	29.8 (21.7)	28.1 (21.5)	28.3 (21.8)
	> 5 sick leave days at baseline	278	56.9 (25.3)	36.9 (23.1)	35.7 (23.2)	37.3 (23.7)
WPAI total work productivity impairment in past 7 days, %	All patients	536	54.8 (27.1)	35.2 (25.9)	32.6 (24.3)	33.6 (25.1)
	0–5 sick leave days at baseline	324	47.5 (25.4)	30.3 (22.8)	28.9 (22.5)	28.6 (23.2)
	> 5 sick leave days at baseline	212	66.0 (25.9)	41.8 (28.4)	37.9 (25.8)	39.8 (26.1)

Data are reported as mean (standard deviation)

*WPAI* Work Productivity and Activity Impairment

^a^Number of patients with data available at baseline for the indicated outcome. Patient numbers at subsequent time points were within 10% of the baseline number

**Fig. 1:**
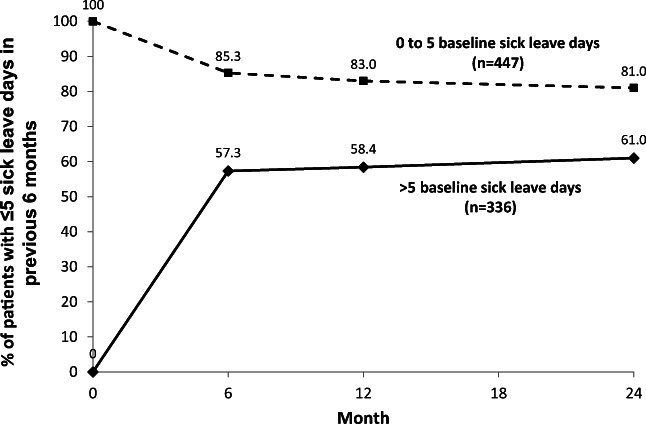
Proportion of patients who achieved normal sick leave duration (≤ 5 days in the previous 6 months) during adalimumab treatment by baseline sick leave days

Of patients who had > 5 days of sick leave in the past 6 months at baseline (*n* = 336), 61% achieved normal sick leave duration (≤ 5 days) during 24 months of adalimumab treatment (Fig. 1). The mean number of sick leave days in this subgroup decreased from 33.7 days in the past 6 months at baseline to 11.1 days at month 24 (Table 2). Improvements in sick leave days were observed at the earliest visit (month 6) and were maintained through month 24. Of patients with ≤ 5 sick leave days at baseline (*n* = 447), more than 80% maintained normal sick leave duration at subsequent visits (Fig. 1).

Improvements were observed in WPAI absenteeism, presenteeism, and total work productivity impairment in all cohorts (Table 2). Sickness absence days reflected by the WPAI absenteeism measure were broadly comparable with self-reported sick leave days in the past 6 months. For instance, for the full patient cohort, assuming a 35-h work week of five 7-h days, 8.4% missed work hours in the previous 7 days (absenteeism in the full patient cohort at month 24) would equal 2.94 h in the past week and 70.56 h (10.08 days) in the past 6 months (24 weeks), which is in the same general range as 7.4 mean sick leave days at month 24. Although presenteeism also improved, the rate of presenteeism in the full patient cohort at month 24 remained relatively high (32.2% compared with 49.9% at month 0).

**Fig. 2:**
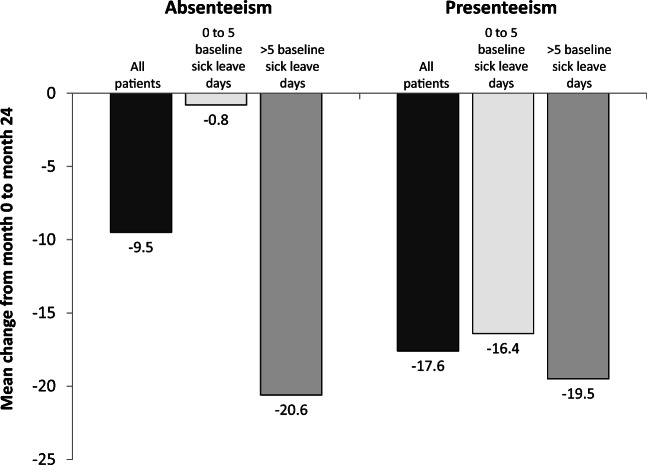
Mean change from baseline (month 0) to month 24 in absenteeism (% of missed work hours) and presenteeism (% of impairment at work) as assessed by the Work Productivity and Activity Impairment questionnaire

As might be expected, the greatest improvements in absenteeism occurred in the subgroup with higher baseline sick leave. In contrast, improvements in presenteeism were similar in the 2 subgroups (Fig. 2).

### Change in disease activity during adalimumab therapy

Both objective (DAS28, tender joint count, swollen joint count) and subjective (HAQ-DI, pain, fatigue, patient global health) measures of disease activity showed marked improvements during adalimumab therapy (Table 3). At month 24, objective measures were comparable in the 2 subgroups. However, subjective measures at month 24 were higher in the subgroup with > 5 days of sick leave at baseline compared with the subgroup with ≤ 5 days of sick leave.

**Table 3:** Disease activity during adalimumab therapy in employed patients

Outcome	Patient population	*n*^a^	Month			
			0	6	12	24
DAS28	All patients	783	4.9 (1.1)	3.0 (1.2)	2.9 (1.2)	2.9 (1.3)
	0–5 sick leave days at baseline	447	4.8 (1.0)	2.9 (1.2)	2.9 (1.2)	2.8 (1.3)
	> 5 sick leave days at baseline	336	5.1 (1.1)	3.2 (1.3)	2.9 (1.2)	3.0 (1.3)
Tender joint count	All patients	783	7.8 (5.8)	2.6 (4.2)	2.1 (3.6)	2.3 (4.2)
	0–5 sick leave days at baseline	447	7.0 (5.4)	2.2 (3.6)	2.0 (3.5)	2.1 (4.1)
	> 5 sick leave days at baseline	336	8.9 (6.2)	3.3 (4.8)	2.1 (3.6)	2.6 (4.3)
Swollen joint count	All patients	783	5.5 (4.9)	1.5 (2.7)	1.3 (2.3)	1.4 (3.1)
	0–5 sick leave days at baseline	447	5.1 (4.5)	1.5 (2.6)	1.4 (2.4)	1.5 (3.3)
	> 5 sick leave days at baseline	336	6.0 (5.2)	1.6 (2.9)	1.2 (2.2)	1.3 (2.8)
HAQ-DI	All patients	780	0.98 (0.63)	0.63 (0.61)	0.61 (0.60)	0.63 (0.63)
	0–5 sick leave days at baseline	446	0.84 (0.60)	0.51 (0.54)	0.52 (0.56)	0.54 (0.59)
	> 5 sick leave days at baseline	334	1.16 (0.63)	0.78 (0.66)	0.73 (0.63)	0.75 (0.66)
Pain^b^	All patients	782	5.6 (2.3)	3.4 (2.3)	3.3 (2.3)	3.3 (2.4)
	0–5 sick leave days at baseline	447	5.2 (2.3)	3.1 (2.2)	3.1 (2.2)	3.1 (2.4)
	> 5 sick leave days at baseline	335	6.0 (2.2)	3.8 (2.3)	3.5 (2.3)	3.6 (2.4)
Fatigue^b^	All patients	783	5.6 (2.7)	3.6 (2.5)	3.6 (2.6)	3.6 (2.7)
	0–5 sick leave days at baseline	447	5.2 (2.7)	3.2 (2.4)	3.3 (2.5)	3.3 (2.6)
	> 5 sick leave days at baseline	336	6.1 (2.4)	4.2 (2.5)	3.9 (2.8)	4.1 (2.7)
Patient global health^b^	All patients	783	5.7 (2.2)	3.5 (2.2)	3.4 (2.2)	3.4 (2.3)
	0–5 sick leave days at baseline	447	5.5 (2.2)	3.2 (2.1)	3.2 (2.2)	3.2 (2.3)
	> 5 sick leave days at baseline	336	6.0 (2.3)	3.9 (2.3)	3.7 (2.3)	3.8 (2.4)

Data are reported as mean (standard deviation)

*DAS28* Disease Activity Score-28 joints, *HAQ-DI* Health Assessment Questionnaire-Disability Index

^a^Number of patients with data available at baseline for the indicated outcome. Patient numbers at subsequent time points were within 10% of the baseline number

^b^Assessed on an 11-point categorical scale from 0 (best) to 10 (worst)

### Predictors of sick leave days

Regression models identified three variables that predicted higher baseline sick leave days: higher baseline HAQ-DI (lower functional levels), previous joint surgery, and male sex (*p* < 0.0001 for all variables). Because most men worked full-time, we considered the possibility that the association with male sex reflected the greater number of days worked by male patients. However, employment level (full-time/part-time) was not identified as a predictor of baseline sick leave days, either with or without the inclusion of sex in the regression model. For change in sick leave days during treatment, more baseline sick leave days in the past 6 months (*p* < 0.0001) and higher levels of pain at baseline (*p* = 0.0007) predicted a greater decrease in the number of sick leave days between baseline and month 6.

## Discussion

Given the chronic nature of RA, the impact of long-term effective therapy on employment-related outcomes is important not only to patients with this disease but to society as a whole. The impact of RA on work productivity in Germany has been well documented. About 20% of German patients with RA are on disability pension [8]. In the employed population, approximately 22% of all sick leave days are due to musculoskeletal disorders, including RA [22]. Total indirect costs due to work disability and sick leave days range from an estimated €3077 to €9754 for each RA patient in Germany; sick leave costs alone account for an estimated €1525 per patient per year [8].

In this observational study, we evaluated data from a cohort of German patients who were employed, either part-time or full-time, at the time of adalimumab initiation, and remained employed during 24 months of adalimumab treatment. This population was chosen to examine the long-term impact of effective therapy on employment outcomes; for this reason, we excluded patients who discontinued treatment, as other studies have previously addressed work outcomes across the broad population of RA patients. [4, 23] Baseline characteristics indicated that the cohort had moderate to severe RA, with a mean disease duration of approximately 8 years. Forty-three percent had higher than normal sick leave at baseline (> 5 days in previous 6 months). Therapy with adalimumab was associated with marked reductions in the number of sick leave days in the past 6 months; mean sick leave days decreased by approximately 50% (from 14.8 to 7.4 days) during 2 years of treatment. For patients with < 5 sick leave days at baseline, there was a small increase in sick leave days over 24 months, although mean sick leave days remained at normal levels. This increase may reflect the progressive nature of RA. Of patients who entered the study with > 5 sick leave days in the previous 6 months, 57% returned to normal sick leave values within 6 months of adalimumab treatment and 61% achieved normal sick leave duration by 24 months. Improvements in the past 7 days were observed in missed hours of work (absenteeism), on-the-job effectiveness (presenteeism), and overall work impairment as measured by the WPAI. Changes in absences and work productivity were reported at the earliest visit and were maintained over 2 years in patients remaining on adalimumab therapy.

Although we observed a reduction in the number of sick leave days and absenteeism during therapy, our data point to reduced productivity at work (presenteeism) as a continuing issue in patients with RA, even during effective treatment. This is in agreement with other reports [7, 24]; one study found that presenteeism was higher than absenteeism in patients with RA and approximately twice as high as in control patients (49% vs 25%; *p* < 0.001) [7]. However, it should be noted that presenteeism rates in patients with RA vary substantially among different countries, ranging from 13.1% in Venezuela to 34.0% in Morocco in one international study, and appear to be influenced by various societal factors, including educational level, standard of living, employment rates, and social protection expenditures [10]. The solution to the issue of presenteeism in RA remains unclear. A study in the Netherlands examined the impact of an intensive targeted intervention program, including case management by a multidisciplinary team, on work productivity in employed patients with RA and difficulties in functioning at work [25]. At the 12-month follow-up, the intervention group did not show a significant improvement in presenteeism compared with standard care. It is possible that other forms of disease management, including targeted management of difficult-to-treat symptoms such as pain or fatigue, might prove more successful in increasing work productivity.

In our study, improvements in work outcomes were accompanied by sustained improvements in disease activity. At 24 months, objective measures were fairly comparable between the 2 subgroups based on baseline sick leave days, whereas subjective measures, including function (HAQ-DI), pain, fatigue, and patient global health, continued to show clear differences. We have previously reported on the critical role of pain and fatigue during RA therapy [26]. It is possible that the higher levels of pain and fatigue reported by patients with > 5 baseline sick leave days may help explain the increased presenteeism in this subgroup at month 24. This hypothesis is consistent with a recent report in which patients whose global assessment scores were higher than their physician global assessment scores (indicating that the patient considered their health status to be worse than the physician did) had more pain, fatigue, and work impairment than patients with concordant assessments [27]. Together with the data reported here, these findings suggest that parameters of patient health that are not fully captured by objective assessments can have an important influence on work ability and productivity.

Other studies of adalimumab have also supported the positive effect of this therapy on reducing work loss in patients with RA. A post hoc analysis from a randomized trial in patients with early RA found significant improvements in WPAI presenteeism and overall work impairment in those receiving adalimumab plus methotrexate compared with placebo plus methotrexate over 26 weeks [14]. Additional short-term (24- to 48-week) studies have documented the effect of adalimumab on reducing missed work days and improving work productivity in Saudi Arabia [12] and Japan [15]. In an earlier study in German patients treated with adalimumab for 12 months, Krüger et al. [11] reported a more modest decrease in sick leave days than observed in our study cohort. The population in that study had more severe disease (DAS28 of 5.6 versus 4.9) and a longer disease duration (9.3 versus 7.8 years) than the cohort assessed here, supporting the authors’ hypothesis that early effective therapy may have a more profound effect on reductions in sickness absence than delayed intervention [11]. Although it has been reported that early intervention improves patient function and reduces employment-related productivity costs [28, 29], more research is needed on the effect of early therapy on work-related outcomes, including presenteeism.

Predictors of an increased number of baseline sick leave days were functional impairment (higher HAQ-DI) at baseline, previous joint surgery, and male sex, while predictors of change in sick leave from baseline to month 6 were a higher number of baseline sick leave days and higher baseline pain scores. The role of sex in sick leave remains unclear. Although male sex has been identified as a predictor of sick leave absence in some studies, including ours, others have found an association between women and sick leave or no association with either sex [9]. The influence of poor physical functioning and pain on sick leave appears to be more consistent across different studies [9]. A recent study of a Swedish biologics registry found that patient-reported outcomes were stronger predictors of subsequent sick leave than more objective measures, such as joint counts or inflammatory markers [30]. Although increased morning stiffness has been associated with employment impairment [31, 32], our regression model did not identify the duration of morning stiffness as a predictor of sick leave days. We note with interest the recent article reporting an association between low income and higher rates of sick leave in German patients with RA [33]. Our observational study did not collect data on patient income so we were unable to analyze this potential predictor.

Our study has limitations, including the use of self-reported measures for work outcomes. Missed work days due to sickness were based on patient recall of events occurring over the previous 6 to 12 months. This time lag may have made it difficult for patients to accurately report these outcomes. However, a study of German patients with RA found that patient-reported data on days of sick leave were comparable with insurance claims data from payers in Germany over six 3-month periods, suggesting that patient recall may be reliable for this outcome [6]. Because patients were only asked about sick leave absences for which they had received a doctor’s certificate, sick leave absences of 3 days or fewer were generally not included, which may have resulted in an underestimation of the number of RA-related sick leave days throughout the study. The similarity between the number of missed work days based on patient recall of sick leave days in the past 6 months and missed work days calculated from WPAI absenteeism in the past 7 days suggests that any underestimation was relatively minor.

Our conclusions are limited to patients who remained on adalimumab therapy for 24 months. Patients who did not benefit from adalimumab may have discontinued from the study before 24 months, which would have excluded them from these analyses as we could no longer follow this patient population given the design of our study. Finally, because the goal of this study was to examine the effect of adalimumab in patients who remained employed during the 2-year study, our analyses did not include patients who left the work force during adalimumab treatment. This is an interesting subset of patients that should be addressed in future studies.

Although the lack of a comparator group can limit the conclusions drawn from observational studies, we believe that in this case, the observational nature of our cohort provides a more realistic picture of employment-related outcomes in the overall RA population than can be obtained from randomized trials. Recent reports indicate that fewer than half of patients in observational cohorts (3.7% to 44%, depending on the study) would meet eligibility criteria for randomized trials of biologic agents [34, 35]. Furthermore, trial-eligible patients have better treatment responses than non-eligible patients, suggesting that the criteria involved in selection may also predispose these patients to better outcomes than the overall RA population [34]. Because patients in our study were not excluded for comorbidities, concomitant medications, or other characteristics that can lead to omission from randomized trials, our data represent the “real-world” long-term impact of adalimumab therapy on sick leave and work productivity across the German population of patients with RA.

In conclusion, this observational study of German patients initiating therapy with adalimumab found marked improvements in employment-related outcomes in employed patients with RA during 2 years of treatment. These improvements were accompanied by reductions in disease activity by both objective and subjective measures. Despite the overall success of therapy, however, a relatively high level of presenteeism, pain, and fatigue remained after 24 months of treatment. It is possible that pain and fatigue contribute to reduced productivity at work, and that targeted management of these symptoms, including exploration of possibly contributory comorbidities, such as osteoarthritis, cardiovascular disease, or depression, may help improve patient global health and work-related outcomes.

## Data Availability

The datasets used in this study are available from the corresponding author on reasonable request.
