# Correction to: Sustained improvement in work outcomes in employed patients with rheumatoid arthritis during 2 years of adalimumab therapy: an observational cohort study

**DOI:** 10.1007/s10067-020-05168-3

**Published:** 2020-05-30

**Authors:** Frank Behrens, Hans-Peter Tony, Michaela Koehm, Eva C. Schwaneck, Holger Gnann, Gerd Greger, Harald Burkhardt, Marc Schmalzing

**Affiliations:** 1 Division of Rheumatology, University Hospital Frankfurt, Goethe University, Frankfurt am Main, Germany; 2 Project Group Translational Medicine & Pharmacology TMP, Fraunhofer Institute for Molecular Biology and Applied Ecology IME, Frankfurt am Main, Germany; 3 Schwerpunkt Rheumatologie/Klinische Immunologie Medizinische Klinik und Poliklinik II, Universität Würzburg, Oberdürrbacher Strasse 6, Würzburg, Germany; 4 Abteilung Biostatistik, GKM Gesellschaft für Therapieforschung mbH, Munich, Germany; 5 AbbVie Deutschland GmbH & Co. KG, Wiesbaden, Germany

**Correction to: Clinical Rheumatology**

10.1007/s10067-020-05038-y

The article “Sustained improvement in work outcomes in employed patients with rheumatoid arthritis during 2 years of adalimumab therapy: an observational cohort study”, written by Frank Behrens, Hans-Peter Tony, Michaela Koehm, Eva C. Schwaneck, Holger Gnann, Gerd Greger, Harald Burkhardt and Marc Schmalzing, was originally published electronically on the publisher’s internet portal on 23 March 2020 without open access. With the author(s)’ decision to opt for Open Choice the copyright of the article changed on 30 May 2020 to © The Author(s) 2020 and the article is forthwith distributed under a Creative Commons Attribution 4.0 International License (https://creativecommons.org/licenses/by/4.0/), which permits use, sharing, adaptation, distribution and reproduction in any medium or format, as long as you give appropriate credit to the original author(s) and the source, provide a link to the Creative Commons license, and indicate if changes were made.

